# Exploring the Potential of Antibiotic Production From Rare Actinobacteria by Whole-Genome Sequencing and Guided MS/MS Analysis

**DOI:** 10.3389/fmicb.2020.01540

**Published:** 2020-07-15

**Authors:** Dini Hu, Chenghang Sun, Tao Jin, Guangyi Fan, Kai Meng Mok, Kai Li, Simon Ming-Yuen Lee

**Affiliations:** ^1^School of Ecology and Nature Conservation, Beijing Forestry University, Beijing, China; ^2^Department of Civil and Environmental Engineering, Faculty of Science and Technology, University of Macau, Macau, China; ^3^Institute of Medicinal Biotechnology, Chinese Academy of Medical Sciences and Peking Union Medical College, Beijing, China; ^4^Beijing Genomics Institute, Shenzhen, China; ^5^State Key Laboratory of Quality Research in Chinese Medicine, Institute of Chinese Medical Sciences, University of Macau, Macau, China

**Keywords:** rare actinobacteria, antibiotics, mangroves, whole-genome sequencing, mass spectrometry

## Abstract

Actinobacteria are well recognized for their production of structurally diverse bioactive secondary metabolites, but the rare actinobacterial genera have been underexploited for such potential. To search for new sources of active compounds, an experiment combining genomic analysis and tandem mass spectrometry (MS/MS) screening was designed to isolate and characterize actinobacterial strains from a mangrove environment in Macau. Fourteen actinobacterial strains were isolated from the collected samples. Partial 16S sequences indicated that they were from six genera, including *Brevibacterium*, *Curtobacterium*, *Kineococcus*, *Micromonospora*, *Mycobacterium*, and *Streptomyces*. The isolate sp.01 showing 99.28% sequence similarity with a reference rare actinobacterial species *Micromonospora aurantiaca* ATCC 27029^T^ was selected for whole genome sequencing. Organization of its gene clusters for secondary metabolite biosynthesis revealed 21 clusters encoded to antibiotic production, which is higher than other *Micromonospora* species. Of the genome-predicted antibiotics, kanamycin was found through guided MS/MS analysis producible by the *M. aurantiaca* strain for the first time. The present study highlighted that genomic analysis combined with MS/MS screening is a promising method to discover potential of antibiotic production from rare actinobacteria.

## Introduction

Microorganisms such as actinobacteria are major sources of antibiotic compounds (Sanchez and Demain, 2015). Actinobacteria are gram-positive bacteria with high GC content in their DNA. They are prolific sources of many well-known antibiotics such as erythromycin, kanamycin, streptomycin, tetracycline, and vancomycin (Schatz et al., 1944; Umezawa et al., 1957; Darken et al., 1960; Cortes et al., 1990; Jung et al., 2007). Among which, nearly 70–80% of the substances are derived from the genus *Streptomyces* (Raja and Prabakarana, 2011). Meanwhile, the non-*Streptomyces* group or rare actinobacteria are lesser known for production of bioactive products due to their lower isolation frequency under laboratory culture conditions (Jose and Jebakumar, 2013). However, recent studies showed that rare actinobacteria could produce antibiotics that were not expected from them; e.g., asukamycin and apramycin were found to be produced by a *Mycobacterium* species (Hu et al., 2019), and rifamorpholines were found to be produced by an *Amycolatopsis* species (Xiao et al., 2017). Thus, the antibiotic-producing potential of the rare actinobacteria is underexplored, and it is not surprising that they are gaining attention (Tiwari and Gupta, 2012; Dhakal et al., 2017; Ishii, 2019).

The antibiotic-producing ability of an actinobacteria is determined by the biosynthetic gene clusters within its genome (Finan, 2017). With the development of molecular sequencing technology, whole genome sequences could provide a new perspective on discovering the potential of antibiotic production from these microbes (Wright, 2019). A microbial genome usually contains 20 to 40 types of biosynthetic gene clusters, each responsible for producing one compound. For example, the widely exploited *Streptomyces avermitilis* harbored 25 gene clusters related to production of type-I polyketide compounds, type-II polyketide-derived compounds, and non-ribosomal peptide synthetases (Ômura et al., 2001; Ikeda et al., 2003). Bacteria with large number of gene clusters within the genome have high potential in synthesizing multiple types of compounds (Challis, 2014). It is known that microbes can exchange genetic information with each other through the process of horizontal gene transfer (Koonin, 2016). A bacteria species habituating in a diverse and rich microbial community therefore has high possibility of having a larger number of gene clusters in its genome (Cooper et al., 2017). A *Streptomyces parvulus* species derived from a mangrove zone was found to have 109 gene clusters, many more than those grown in other natural environments (Hu et al., 2018). Mangroves are known as productive inter-tidal ecosystems with rich microbial communities, having bacteria making up 91% of their total microbial biomass (Azman et al., 2015). Their locations being at the transition area between terrestrial and marine habitats give mangroves the advantage of harboring very diverse bacterial communities from two different environments, providing conditions for microbes to exchange genetic information with each other. Thus, mangrove-derived microbes could have greater genetic potential to synthesize more bioactive substances.

Even when genomic information of a bacterial species is available, many of its gene clusters are expressed only weakly or even silent under laboratory fermentation conditions. To ascertain which secondary metabolites can actually be produced by the tested strain, chemical analysis is necessary. The advancement of instrumentation for analytical chemistry has speeded up the exploration of microbial-derived secondary metabolites. For instance, MALDI-TOF-MS was used to identify 10 secondary metabolites related to previously uncharacterized gene clusters in *Streptomyces hygroscopicus* (Kersten et al., 2011), ESI-IT-MS was applied to uncover a compound of strong antibacterial activity produced by *Gliocladium* sp. (Koolen et al., 2012), and a Q-Trap LC-MS platform was utilized to unravel natural products with antimicrobial activity produced by *Vibrio cholerae* (Sikora et al., 2018). Therefore, the coupled method of genome mining and mass spectrometry could reveal the antibiotic production potential of isolated microbes.

The present study is to explore the potential of antibiotic production of a rare actinobacteria from a mangrove environment. Actinobacterial strains were first isolated from collected plant samples by plate culturing and identified by 16S sequence analysis. Whole-genome sequencing and genomic analysis of a selected strain were performed to identify the gene clusters encoding antibiotic production. Preliminary tandem mass spectrometry (MS/MS) analysis was then used to identify the small molecules produced from the tested strain under laboratory conditions. The results could provide reference information for exploring new sources of antibiotics for further research and application in the medical industry.

## Materials and Methods

### Environmental Sampling

Plant samples from *Aegiceras corniculatum* and *Kandelia candel* mangrove trees were collected at two sites, S1 and S2, of different environmental characteristics in the Cotai City Ecological Reserve in Macau during March and May of 2017 as listed in Table 1. Samples of *K. candel* were only collected from site S2 due to its unavailability at site S1. Several leaves were picked from different parts of one tree and put into a sterile plastic bag as a sample. All specimens were transported back to the laboratory immediately from site.

**TABLE 1:** Locations and mangrove tree types for sampling.

**Sites**	**Coordinate**	**Samples**	**Sample type**
S1	22°8′30″N 113°33′11″E	*Aegiceras corniculatum*	Plant leaves
S2	22°8′29″N 113°33′5″E	*Aegiceras corniculatum, Kandelia candel*	Plant leaves

### Selective Isolation of Actinobacteria Strains

The procedures for plant sample pretreatment and plate culture followed those in Hu et al. (2018). In short, Tween-20, NaClO, NaS_2_O_3_, ethanol, and NaHCO_3_ were used to clean the surfaces of the plant samples. Their homogenates were prepared and then diluted to plate onto isolation media. Seven different media including ISP media 2, ISP media 4, ISP media 7, Gauze No. 1, Nutrient Agar, halothiobacillus HL2, and Czapek were used to culture actinobacterial strains. The culture media were incubated at 28°C for 7–30 days.

### Extraction of DNA From Pure Cultures and PCR Amplification of 16S rRNA

DNA was extracted from each purified isolate for bacterial species identification. The procedures for DNA extraction and PCR amplification followed those in Hu et al. (2018). The universal bacteria primer pair of 27F (5′-AGAGTTTGATCCTGGCTCAG-3′) and 1492R (5′-TACGGCTACCTTGTTACGACTT-3′) were used for amplification of 16S rRNA sequences (Mao et al., 2012). The yielded DNA was then sequenced on the Sanger sequencing platform. The generated sequences were compared with EzBioCloud database to identify the species^1^.

### Genomic Analysis

The procedures for whole genome sequencing and genomic analysis followed those in Hu et al. (2018). Genomic DNA was prepared using the TIANamp Bacteria DNA Kit (TIANGEN Biotech Co. Ltd). The genomic DNA library was constructed using the NEBNext Ultra II DNA Library Prep Kit for sequencing on Illumina NovaSeq HiSeq 4000. Genome assembly and gene prediction were performed by IBDA and MetaGeneMark, respectively. The tRNAscan-SE was used for prediction of ribosomal RNAs (rRNAs). After searching against the Kyoto Encyclopedia of Genes and Genomes (KEGG) database, the functional categories were assigned through the genome. Anti-SMASH (version 4.0 and 5.0) was used to predict the biosynthetic gene clusters for production of secondary metabolites^2^. A threshold of homology similarity at 15% was chosen for analysis based on previous studies (Huang et al., 2016; Othoum et al., 2019) that found that gene clusters with low similarity could contain unique structures. Mega 7.0 was used to construct the phylogenetic tree. The sequence of *Micromonospora aurantiaca* sp.01 generated in this study has been deposited in the GenBank database with the accession number RIBT00000000.

### Crude Extract Preparation and Mass Spectrometric Analysis

The procedures for biochemical screening followed those in Hu et al. (2018). The 4000 Q TRAP LC/MS/MS mass spectrometer system was equipped with a micro-ESI-MS for separation and analysis of the secondary metabolites produced by a targeted strain. Full-scan MS data were acquired from mass-to-charge ratios (m/z) between 100 and 1000 at an acquisition rate of 0.6 s per spectrum. ESI source was operated in the positive mode at 3.0 kV of capillary voltage and 20 V of cone voltage. Kanamycin standard sulfate salt was purchased from Sigma (St. Louis MO, United States). After drying in vacuum at 70°C for 3 h, individual stock solutions were prepared at a concentration of 50 mg/ml in water (Turnipseed et al., 2009).

## Results

### Isolation and Identification of Endophytic Actinobacterial Strains

**FIGURE 1:**
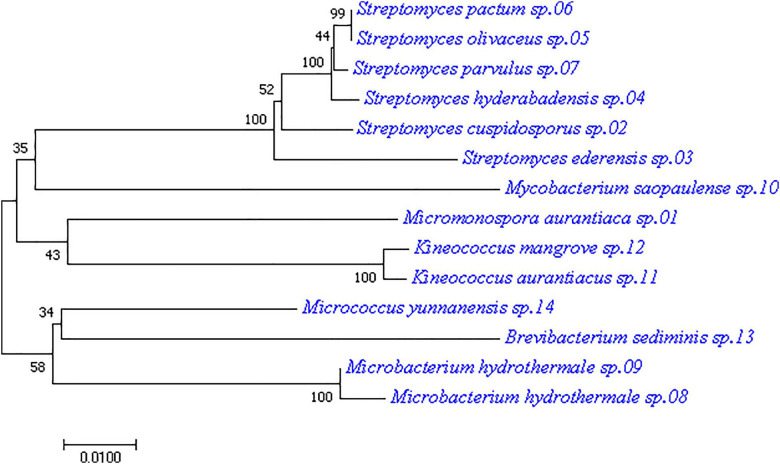
Phylogenetic tree of the 14 actinobacterial strains isolated from mangrove leaves. The tree was constructed with the neighbor-joining method having the number of bootstrap replications set to 1000.

Culturable bacteria from the mangrove plant samples were analyzed by plating their pre-treated homogenates onto seven different isolation media. The 16S rRNA sequences of the isolated single colonies were amplified by PCR to recover the needed fragments for subsequent Sanger sequencing. The results were then analyzed by using EzbioCloud server referencing with the NCBI database for species identification. The resulting partial 16S sequences revealed 71 endophytic bacterial strains from the plant samples (Supplementary Table S1). Among them, 14 strains were from six genera of the actinobacteria class, including *Brevibacterium*, *Kineococcus*, *Microbacterium*, *Micromonospora*, *Mycobacterium*, and *Streptomyces*. They are listed in Table 2 together with their reference strains and corresponding 16S rRNA gene sequence similarity percentages. Applying the 97.0% similarity threshold widely used for novel bacterial species identification (Sripreechasak et al., 2013; Kim et al., 2014) as a reference, there were seven isolates considered highly similar to their reference strains (similarity > 99%), 6 isolates considered closely related to their reference strains (similarity > 98%), and 1 isolate considered between closely related to and related to its reference strain (similarity = 98%). In terms of genus diversity, *Streptomyces* was the dominant one harboring six strains, followed by *Microbacterium* with three strains, and *Kineococcus* with two strains. Meanwhile, each of the genus *Brevibacterium*, *Micromonospora*, and *Mycobacterium* had 1 strain. A phylogenetic tree was constructed for these 14 actinobacterial isolates to demonstrate their evolutionary phylogenetic relationship (Figure 1).

**TABLE 2:** The endophytic actinobacterial community identified based on the partial 16S rRNA sequences.

**Isolate**	**Top-hit taxon at species level**	**Length (bp)**	**Similarity (%)**
01	*Micromonospora aurantiaca* ATCC 27029^T^	942	99.28
02	*Streptomyces cuspidosporus* NBRC 12378^T^	1005	98.28
03	*Streptomyces ederensis* NBRC 15410^T^	930	98.79
04	*Streptomyces hyderabadensis* OU-40^T^	1001	99.18
05	*Streptomyces olivaceus* NRRL B-3009^T^	1005	99.25
06	*Streptomyces pactum* NBRC 13433^T^	930	99.57
07	*Streptomyces parvulus* NBRC 13193^T^	1047	99.13
08	*Microbacterium hydrothermale* 0704C9-2^T^	1045	98.16
09	*Microbacterium hydrothermale* 0704C9-2^T^	1045	98.58
10	*Mycobacterium saopaulense* EPM 10906^T^	1504	99.90
11	*Kineococcus aurantiacus* IFO 15268^T^	972	98.20
12	*Kineococcus mangrove* L2-1-L1^T^	1048	98.00
13	*Brevibacterium sediminis* FXJ8.269^T^	1005	98.90
14	*Micrococcus yunnanensis* YIM 65004^T^	850	99.56

As only the genus *Streptomyces* has been exploited extensively for antibiotic production over the years, the underexploited non-*Streptomyces* or rare actinobacteria group could have much potential not being discovered even for their better-known genera. Among the rare actinobacteria, the genus *Micromonospora* has been found predominant in mangroves of different parts of the world over the years (Azman et al., 2015); it was also found in the present study. The phylogenetic tree in Figure 1 showed that isolate *M. aurantiaca* sp.01 was located on the same tree branch of isolate 11 and isolate 12 of the *Kineococcus* genus, suggesting that they were closely related. It also had close relation with the *Streptomyces* genus isolates 02, 03, 04, 05, 06, and 07 as well as with the *Mycobacterium* genus isolate 10. Meanwhile, isolate *M. aurantiaca* sp.01 was found to have farther genetic distances with isolates 08, 09, 13, and 14 of the genera *Microbacterium*, *Brevibacterium*, and *Micrococcus*. Finally, isolate *M. aurantiaca* sp.01 that exhibited 99.28% 16S similarity with the reference strain *M. aurantiaca* ATCC 27029^T^ was selected for exploration of its bioactive potential through whole genome sequencing and chemical analysis.

### General Feature of the Genome and Associated Secondary Metabolome

**FIGURE 2:**
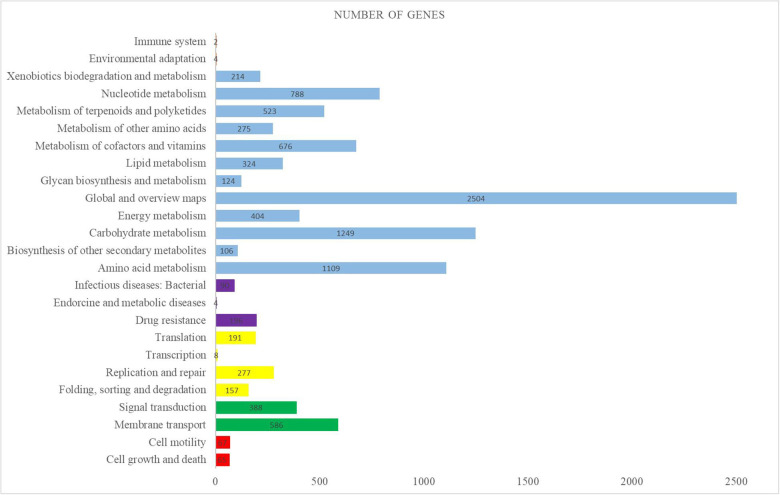
Distribution of KEGG pathways in the *Micromonospora aurantiaca* sp.01 genome. The distribution of the predicted functional classification proteins was assigned by comparison with the KEGG database. Figure displays the top five KEGG Orthology (KO) categories of the assigned sequences, including cellular process in red, genetic information processing in yellow, environmental information processing in green, organismal systems in orange, human diseases in purple, and metabolism in blue.

The complete genome sequence of the *M. aurantiaca* sp.01 was deposited to NCBI that produced 16,952,140 reads and 914 scaffolds. The *De novo* assembly was then done on the yielded reads by using IDBA and generated a total consensus of 8,186,173 bp, with an average size of 8956 bp and a G + C content of 72.31%. Gene predictions from the genome of *M. aurantiaca* sp.01 were performed through annotation by comparison with the KEGG database. It showed that a total of 7226 protein-encoding genes were conserved in the genome, 72 tRNA were predicted, the average CDS length was 969 bp, and the coding density was about 89.56%. The predicted proteins assigned in the KEGG pathways revealed that the top three categories of functional classification were “global and overview maps, carbohydrate metabolism, and amino acid metabolism” (Figure 2).

### Antibiotic Biosynthetic Capability of *M. aurantiaca* sp.01

AntiSMASH was used to get a preview of the secondary metabolome in the genome of the *M. aurantiaca* sp.01 based on its sequence analysis. Biosynthetic gene clusters encoding for known domains of polyketides (PKS), non-ribosomal peptides (NRPS), as well as core and accessory genes were identified by searching individual open reading frames (ORFs) against the sequence database. Nucleotide sequence analysis found 77 gene clusters related to secondary metabolite biosynthesis in the *M. aurantiaca* sp.01 genome. They were predicted to be involved in PKS, NRPS, bacteriocin, fatty acid, lantipeptide, oligosaccharide, saccharide, siderophore, terpene, and many putative products (Table 3). Among them, 42 gene clusters were annotated to 37 known secondary metabolites as antibiotics, bioactive compounds, and other products.

**TABLE 3:** Overview of the predicted secondary metabolites from biosynthetic gene clusters of the *Micromonospora aurantiaca* sp.01 detected by anti-SMASH.

**Type**	**Location**	**Category**	**Predicted product**	**Similarity (%)**	**Reference strain**	**Accession number**
Type I PKS	1-5012	Antibiotics	Aculeximycin	23	*Kutzneria albida* DSM 43870	CP007155
Type I PKS	1-14106		Amphotericin	23	*Streptomyces nodosus*	AF357202
Type I PKS	1-37928		Chalcomycin	12	*Streptomyces bikiniensis* strain NRRL 2737	AY509120
Type I PKS	1-6925		Lobophorin	13	*Streptomyces* sp. SCSIO 01127	KC013978
Type I PKS	1-27275		Naphthomycin	25	*Streptomyces* sp. CS	GQ452266
Type I PKS	1-10526		Naphthomycin	15	*Streptomyces* sp. CS	GQ452266
Type I PKS	1-10185		Nigericin	44	Not derived from GenBank	
Type I PKS-NRPS	57831-125457		Bleomycin	12	*Streptomyces verticillus*	AF210249
Type II PKS	6402-33702		Kosinostatin	11	*Micromonospora* sp. TP-A0468	JN038178
Type II PKS-Fatty acid	66691-116600		Xantholipin	16	*Streptomyces flavogriseus* strain SIIA-A02191	GQ421798
NRPS-Type I PKS-Siderophore-Otherks	30808-150128		Azicemicin	13	*Kibdelosporangium* sp. MJ126-NF4	GU134622
Lantipeptide	54973-80539		Incednine	2	*Streptomyces* sp. ML694-90F3	AB767280
Oligosaccharide	669851-709255		Cosmomycin D	35	*Streptomyces olindensis* strain DAUFPE 5622 2	JJOH01000002
Terpene	118010-138340		Nocathiacin	4	*Nocardia* sp. ATCC 202099	GU564398
Putative	120901-132867		Clorobiocin	14	*Streptomyces roseochromogenes* subsp. oscitans	AF329398
Putative	224076-232740		Fengycin	20	*Bacillus amyloliquefaciens* subsp. plantarum str. FZB42	CP000560
Putative	383554-397823		Friulimicin	6	*Actinoplanes firuliensis*	AJ488769
Putative	121404-131026		Kanamycin	15	*Streptomyces kanamyceticus*	AB254081
Putative	16035-35841		Lasalocid	3	*Streptomyces lasaliensis* strain NRRL 3382R	FM173265
Putative	223676-244438		Maklamicin	8	*Micromonospora* sp. GMKU326	LC021382
Putative	69108-79942		Pyrrolomycin	18	*Streptomyces* sp. UC 11065	EF140903
Putative	454-16503		Rifamycin	15	*Salinispora arenicola* CNS-205	CP000850
Type I PKS	1-17368	Bioactive compounds	Halstoctacosanolide	77	*Streptomyces halstedii*	AB241068
Type I PKS	1-5987		Stambomycin	36	*Streptomyces ambofaciens* ATCC 23877	AM238664
Type I PKS	169785-215637		Tiancimycin	19	*Streptomyces* sp. CB03234	KT716443
Fatty acid	188064-209008		Chlorizidine A	7	*Streptomyces* sp. CNH-287	KF585133
Oligosaccharide-Fatty acid-Terpene-NRPS	298906-371903		Lobosamide	13	*Micromonospora* sp. RL09-050-HVF-A	KT209587
Transatpks-NRPS-Otherks	514354-599495		Leinamycin	15	*Streptomyces atroolivaceus*	AF484556
Putative	408661-429833		Diazepinomicin	5	*Micromonospora* sp. M42	KK037233
Type I PKS	1-6472	Others	ECO-02301	35	*Streptomyces aizunensis* strain NRRL B-11277	AY899214
Type I PKS	219687-252289		ECO-02301	42	*Streptomyces aizunensis* strain NRRL B-11277	AY899214
Type III PKS	532-41533		Alkyl-O-Dihydrogeranyl-Methoxyhydroquinones	71	*Actinoplanes missouriensis*	AP012319
Bacteriocin-Terpene	19400-48982		Lymphostin	33	*Salinispora* tropica CNB-440	CP000667
Other	1-10874		Landepoxcin	11	Uncultured bacterium AR 412	KP830093
Saccharide	144044-173388		Clavulanic acid	8	*Streptomyces clavuligerus* ATCC 27064	DS570624
Saccharide	98895-137389		Echosides	17	*Streptomyces* sp. LZ35	KJ156360
Saccharide	8423-64174		Phosphonoglycans	6	*Stackebrandtia nassauensis* DSM 44728	CP001778
Saccharide	192353-255024		Phosphonoglycans	12	*Glycomyces* sp. NRRL B-16210	KJ125437
Terpene	8286-21936		Phosphonoglycans	3	*Glycomyces* sp. NRRL B-16210	KJ125437
Terpene	388501-409427		Sioxanthin	100	*Salinispora tropica* CNB-440	CP000667
Putative	576-23953		Galbonolides	10	*Streptomyces galbus* strain KCCM 41354	GU300145
Putative	11934-29777		Sioxanthin	100	*Salinispora tropica* CNB-440	CP000667
Type I PKS	1-5290					
Type I PKS	1-3407					
Type I PKS	1-1770					
NRPS	1-12378					
NRPS	1-3469					
Lantipeptide	4637-27306					
Lantipeptide	17353-42750					
Saccharide	315838-339960					
Saccharide	181740-203951					
Siderophore	35604-48815					
Terpene	44170-65120					
Transatpks-Type I PKS-NRPS	1-41316					
Putative	377809-386812					
Putative	492675-511199					
Putative	107942-124615					
Putative	45518-54802					
Putative	82295-91798					
Putative	239829-245173					
Putative	322642-334528					
Putative	378553-385023					
Putative	80319-89810					
Putative	143388-158622					
Putative	4322-12058					
Putative	197891-207426					
Putative	11-12526					
Putative	14154-35274					
Putative	104335-113228					
Putative	10649-33913					
Putative	48858-73536					
Putative	52917-72592					
Putative	17929-23324					
Putative	26432-38927					
Putative	448-24154					
Putative	742-14198					
Putative	3-13726					

Twenty-one of these 37 secondary metabolites were antibiotics with 10 associated with PKS/NRPS pathways. It is known that secondary metabolite produced through the PKS/NRPS pathway is one of the most important biosynthesis processes involved with the chemical synthesis of biologically active compounds produced by microorganisms (Ansari et al., 2004). Genome analysis of the *M. aurantiaca* sp.01 showed that it contained at least 23 kinds of PKS/NRPS gene clusters, but only 11 of them were found related to known antibiotics.

**FIGURE 3:**
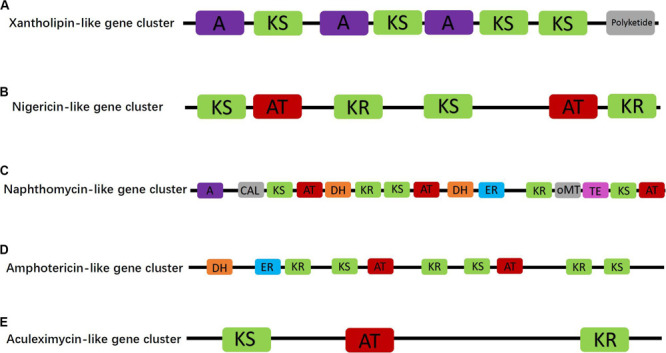
Proposed antibiotic biosynthetic gene clusters of xantholipin-like **(A)**, nigericin-like **(B)**, naphthomycin-like **(C)**, amphotericin-like **(D)**, and aculeximycin-like **(E)** compound in the *Micromonospora aurantiaca* sp.01. Domain notation: A, adenylation; KS, β-ketoacyl synthase; AT, acyl-CoA/ACP transacylase; DH, dehydratase; TE, thioesterase; KR, ketoreductase; CAL, CoA ligase; ER, enoyl reductase; oMT, O-methyltransferase.

Furthermore, five of the PKS/NRPS gene clusters with homology similarity greater than 15% were related to synthesis of antibiotic compounds, namely, aculeximycin, amphotericin, naphthomycin, nigericin, and xantholipin. The xantholipin-like cluster was a hybrid type II PKS-fatty acid gene cluster containing 46 ORFs and 8 catalytic domains, one of which is directly related to polyketide synthetase (Figure 3A). Core biosynthetic genes of this cluster were annotated to cytochrome P450, AMP-dependent synthetase and ligase, beta-ketoacyl synthase, and AraC family transcriptional regulator. Additional biosynthetic genes were related to 8-amino-7-oxononanoate synthase, aminotransferase, alpha/beta hydrolase domain-containing protein, HAD-superfamily hydrolase, subfamily IA, variant, 1-deoxy-D-xylulose-5-phosphate synthase, transketolase, acyl carrier protein, acetyl-CoA carboxylase biotin carboxylase, carboxyl transferase, polyketide synthesis cyclase, and oxidoreductase. In addition, several transport-related and regulatory genes can also be found in the genome, including major facilitator transporter, RND family efflux transporter MFP subunit, transcriptional regulator, and LuxR family transcriptional regulator.

Besides the hybrid synthase of PKS-type gene cluster, one of the Type I PKS gene clusters contained six catalytic domains and was responsible for nigericin biosynthesis (Figure 3B). In the genome of *M. aurantiaca* sp.01, only two ORFs of core biosynthetic genes, namely, beta-ketoacyl synthase and malonyl CoA-acyl carrier protein transacylase, were annotated to nigericin biosynthesis. No additional biosynthetic genes were found in the nigericin biosynthetic gene cluster. This cluster displayed 44% homology to an existing cluster that was not recorded in the GenBank. Thus, it could play an important role in the synthesis of the nigericin-derived primer unit, meaning that these genes could have been transferred horizontally from other microorganisms.

The other three Type I PKS antibiotic biosynthetic gene clusters were naphthomycin-like, amphotericin-like, and aculeximycin-like. The naphthomycin gene cluster showed 25% homology to that of *Streptomyces* sp. CS and contained 11 ORFs, in turn encoding 15 catalytic domains related to the polyketide synthetase (Figure 3C). Core and additional biosynthetic genes were annotated to AMP-dependent synthetase and ligase, 3-dehydroquinate synthase, monooxygenase FAD-binding, methyltransferase, thioesterase, beta-ketoacyl synthase, and malonyl CoA-acyl carrier protein transacylase. The amphotericin gene cluster also had two ORFs encoded to 10 domains (Figure 3D). Compared with the most similar known cluster of *Streptomyces nodosus*, the amphotericin gene cluster in the studied strain had several unique biosynthetic genes, such as crotonyl-CoA reductase/alcohol dehydrogenase. The last Type I PKS antibiotic synthetic gene cluster was related to aculeximycin synthesis and contained three domains (Figure 3E). Its core biosynthetic genes were annotated for beta-ketoacyl synthase and short-chain dehydrogenase/reductase SDR.

It is worth noting that many observed gene clusters in the *M. aurantiaca* sp.01 displayed similarity to known clusters of non-*Micromonospora* strains, meaning that it could probably synthesize many small-molecule compounds through different pathways.

### Genome-Guided Natural Product Discovery of Kanamycin

With the potential antibiotics and bioactive compounds predicted by the genome analysis, secondary metabolites produced by of the *M. aurantiaca* sp.01 under laboratory conditions were examined by reviewing its small-molecule profile for the presence of the predicted compounds. As fermentation and mass spectrometry were used to identify the predicted compounds that are characterized by their m/z, m/z values of 33 predicted secondary metabolites were first estimated theoretically with ChemDraw as listed in Table 4. As the chemical structures of stambomycin, alkyl-O-dihydrogeranyl-methoxyhydroquinones, landepoxcin, and phosphonoglycans are not known, values of their m/z could not be estimated and are marked as N/A in Table 4. A 4000 Q TRAP LC/MS/MS system was used to separate the secondary metabolites of the *M. aurantiaca* sp.01 for analysis. In the first scan mode, four predicted secondary metabolites were detected in the range of m/z between 400 and 500 as shown in Table 4. Only one was an antibiotic. The other three were bioactive compounds.

**TABLE 4:** Predicted and detected values of m/z for the potential secondary metabolites of the *Micromonospora aurantiaca* sp.01.

**Category**	**Most similar known cluster**	**Theoretical MH^+^ (m/z)**	**Detected MH^+^ (m/z)**
Antibiotics	Aculeximycin	1673.97297	
	Amphotericin	896.50076	
	Chalcomycin	701.37484	
	Lobophorin	1157.63726	
	Naphthomycin	706.27829	
	Nigericin	725.48399	
	Bleomycin	1415.52681	
	Kosinostatin	632.23700	
	Xantholipin	552.06975	
	Azicemicin	460.16076	
	Nocathiacin	1307.18980	
	Incednine	738.46934	
	Cosmomycin D	1189.59070	
	Clorobiocin	697.21642	
	Fengycin	1463.80376	
	Friulimicin	1303.68980	
	Kanamycin	485.24589	485.4
	Lasalocid	591.38970	
	Maklamicin	525.32162	
	Pyrrolomycin	356.91813	
	Rifamycin	698.31765	
Bioactive compounds	Halstoctacosanolide	817.51021	
	Stambomycin	N/A	
	Tiancimycin	463.12672	463.4
	Chlorizidine A	444.94943	
	Lobosamide	484.30630	484.4
	Leinamycin	511.10312	
	Diazepinomicin	463.25968	463.4
Others	ECO-02301	1285.79376	
	Alkyl-O-Dihydrogeranyl-Methoxyhydroquinones	N/A	
	Lymphostin	311.11442	
	Landepoxcin	N/A	
	Clavulanic acid	200.05590	
	Echosides	467.17060	
	Phosphonoglycans	N/A	
	Sioxanthin	727.45738	
	Galbonolides	381.22772	

**FIGURE 4:**
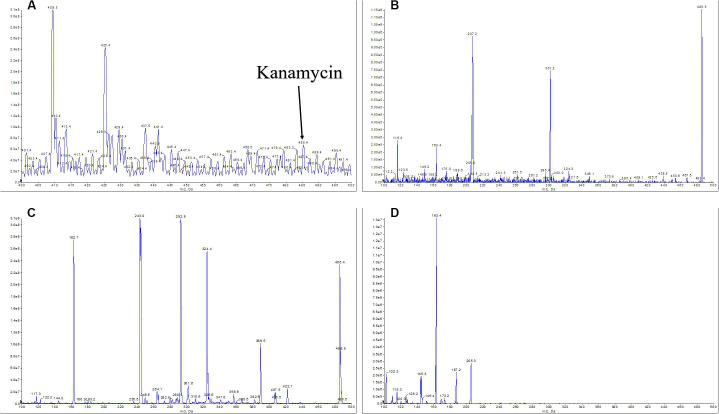
MS/MS analysis of ethyl acetate extraction of the *Micromonospora aurantiaca* sp.01 and a standard kanamycin compound. Based on the mass and fragmentation data, the compound was identified as kanamycin ([M + H]^+^ at m/z = 485.4 **(A)**, in-source fragments at m/z = 163.4 **(B)**). The MS spectra of standard kanamycin were obtained for the ion at [M + H]^+^ at m/z = 485.4 **(C)** and in-source fragments at m/z = 163.4 **(D)**.

However, only the antibiotic kanamycin at m/z = 485.4 (Figure 4A) could be observed in the second scan mode by its corresponding in-source fragment at m/z = 163.4 (Figure 4B) referencing to the value published in Zhang X. et al. (2019). A standard kanamycin compound was also fed to the 4000 Q TRAP LC/MS/MS system to generate its first scan and second scan spectra that gave its m/z = 485.4 (Figure 4C) and m/z = 163.4 (Figure 4D) measurements, respectively. Production of kanamycin by the *M. aurantiaca* sp.01 was confirmed.

## Discussion

Microorganisms are prolific sources of diverse bioactive metabolites for many important natural products applied in agricultural, environmental, medical, and industrial areas (Katz and Baltz, 2016), and the actinobacterial group is extremely important for the pharmaceutical sector (Manivasagan et al., 2015). Among the actinobacteria in natural environments, *Streptomyces* species represent the dominant population; they are commonly found in heavy metal-contaminated soil and their abundances increase with heavy metal stress and the depth of soil (Cao et al., 2017; Zhang et al., 2019b; Wang et al., 2020). As a result, the non-*Streptomyces* species (or rare actinobacteria) are relatively underexplored and less studied due to their low abundance in taxa (Subramani and Aalbersberg, 2013). In this study, there were 71 bacterial species isolated from the plant samples of a mangrove environment in Macau. There were six genera of actinobacteria found in 14 isolates; they were *Brevibacterium*, *Kineococcus*, *Microbacterium*, *Micromonospora*, *Mycobacterium*, and *Streptomyces*. It is known that 70–80% of discovered bioactive compounds are derived from the *Streptomyces* genus (Qian et al., 2015). Because of this, *Streptomyces* strains isolated from different environments were repeatedly found to produce similar chemical compounds (Matsumoto and Takahashi, 2017). As a result, the search for useful antibiotics from other sources such as rare actinobacteria for new natural products is of great interest (Demain, 2014). *Micromonospora* has been reported as a dominant genus of rare actinobacteria in different studied sites (Xu et al., 1996; Ara et al., 2002; Prieto-Davó et al., 2008; Carro et al., 2012), and it was found to produce a commonly used aminoglycoside antibiotic, gentamicin (Weinstein et al., 1963). Meanwhile, several novel natural products such as retymicin, galtamycin B, saquayamycin Z, and ribofuranosyllumichrome have also been isolated successfully from *Micromonospora* (Antal et al., 2005). Therefore, the *M. aurantiaca* sp.01, a rare actinobacterial strain isolated in the present study, was investigated for its biosynthesis potential in depth through whole genome sequencing and mining. Note that no known antibiotic compounds have been reported to be produced by any *M. aurantiaca* strain before (Kim et al., 2006).

Sequenced genomes can provide substantial evidence for the presence of highly diverse secondary metabolic pathways in microorganisms (Bachmann et al., 2014). Gene clusters involved in the synthesis pathways of secondary metabolites in the *M. aurantiaca* sp.01 were identified by complete sequencing, assembly, and annotation of its genome. Genome mining results showed that the species has the potential to produce 21 antibiotics (Table 4). This predicted potential was considerably greater than those observed in previous studies; it was reported before that only several types of fatty acids and amino acids could be identified in the extracts of *M. aurantiaca* strains (Dickschat et al., 2011; Xu et al., 2014). A striking feature of the *M. aurantiaca* sp.01 genome was that it possessed remarkably diverse and abundant PKS biosynthetic pathways; it had 11 PKS gene clusters with similarity over 15% (Table 3). This number was greater than those in other model actinobacterial genomes, e.g., *Salinispora tropica* only had only four PKS gene clusters (Udwary et al., 2007), *Streptomyces ambofaciens* ATCC23877 had 9 (Laureti et al., 2011), and *Sorangium cellulosum* So ce90 also had 9 (Molnar et al., 2000). Another motivation for sequencing the *M. aurantiaca* sp.01 was that it was considered the most abundant rare actinobacterial genus other than the *Streptomyces* spp., and no known antibiotic compounds had been reported from any *M. aurantiaca* strain (Kim et al., 2006). The high genetic potential for secondary metabolite production observed in the *M. aurantiaca* sp.01 genome is encouraging, and more exciting is that most of these predicted antibiotics had never been identified from the *Micromonospora* genus under fermentation cultivation conditions before. On the other hand, these genome-predicted secondary metabolites had been identified widely from other actinobacterial or non-actinobacterial strains, such as *Streptomyces flavogriseus* strain SIIA-A02191, *Streptomyces* sp. CS, *S. nodosus*, *Kutzneria albida* DSM 43870, *Bacillus amyloliquefaciens* subsp. plantarum str. FZB42, *Streptomyces* sp. UC 11065, *Streptomyces kanamyceticus*, *Salinispora arenicola* CNS-205, and *Streptomyces olindensis* strain DAUFPE 5622 2 (Table 3). There is no doubt that the genome-predicted secondary metabolite production patterns of the *M. aurantiaca* sp.01 isolated from mangroves in this study were highly complex.

Mangroves could grow in the harsh conditions of high salinity, extreme tides, strong winds, high temperatures, and muddy anaerobic soils (Ansari et al., 2014). Actinobacterial species such as the *M. aurantiaca* sp.01 of the present study growing in mangroves need to cope with such extreme and biodiverse environment by taking on adaptive strategies that could be reflected in their genome sequences. Previous studies had shown that actinobacterial species in extreme environments had a larger number of genes involved in various types of metabolic pathways when compared with strains from other natural environments (Hu et al., 2018, 2019). Therefore, unexpected abilities in producing novel or more natural products of the actinobacterial strains from extreme environments such as mangroves could be revealed with DNA sequencing technology and genomic analysis.

The assembly, functional annotation, and detailed analysis of the *M. aurantiaca* sp.01 genome sequences were to better understand the biosynthetic potential of a rare actinobacteria. Based on the genome mining results, MS screening was used to look for the predicted secondary metabolites, particularly antibiotics, of the *M. aurantiaca* sp.01 under laboratory conditions. In general, HPLC can accurately and efficiently separate and quantify each component in a mixture; it does involve tedious development of an appropriate analytical method suitable for an unknown (or a partly known) sample (Zhang et al., 2019a). MS analysis, coupled with MS/MS, is efficient and reliable in identifying unknown compounds without the need for a specific method development, which is more suitable for the present study to rapidly identify the compounds from fermentation products. The MS screening results revealed that the predicted secondary metabolite of kanamycin could be produced. Genomic analysis showed that the kanamycin-like biosynthetic gene cluster of the *M. aurantiaca* sp.01 consisted of 11 ORFs with the core biosynthetic genes encoded to short-chain dehydrogenase/reductase SDR and acyl-CoA dehydrogenase. The finding of kanamycin production in the studied strain was exciting as this antibiotic was first isolated from the *S. kanamyceticus* species (Umezawa, 1958), and there were no reports on its production by any *M. aurantiaca* species until now.

In addition, using the MS spectrum of the standard kanamycin at 50 mg/ml mass concentration as a reference (Figures 4C,D), the estimated kanamycin yield of the *M. aurantiaca* sp.01 cultured in 20 ml of medium with glucose as carbon source for 7 days at pH 7.2 was over 2 mg/ml. Studies indicated that varying the culture conditions, such as pH value, and carbon and nitrogen source, of the kanamycin producing *S. kanamyceticus* species would vary its yield. For example, Basak and Majumdar (1973) found that a *S. kanamyceticus* ATCC 12853 strain cultured in 30 ml of medium with glucose as carbon source for 7 days at pH 7.0 yielded a 2 mg/ml kanamycin mass concentration, and then increasing just the pH value to 8.5 boosted the yield up to 39.6 mg/ml (Basak and Majumdar, 1973). In Pandey et al. (2005), different carbon and nitrogen sources were tested for culturing a *S. kanamyceticus* M27 strain and it was found that using dextrose (chemically identical to glucose) as carbon source and NH_4_H_2_PO_4_ (ammonium dihydrogen phosphate) as nitrogen source gave the highest antibiotic production. Thus, the *M. aurantiaca* sp.01 strain of the present study may also increase its kanamycin yield if the culture conditions are adjusted. The *M. aurantiaca* species could potentially be an alternate source of kanamycin. Further study on this is needed.

Another observation is that although the biosynthetic potential of an interested actinobacterial strain is revealed by genomic analysis, expressions of those potentially producible secondary metabolites depend highly on the culture conditions, making many gene clusters remain silent under regular laboratory fermentation conditions. In fact, researches in finding different methods such as homologous and heterologous expression, metabolism remodeling, and metabolic engineering to activate these cryptic genes are very active (Ochi, 2017). Therefore, the present findings also provide evidence for the needs of similar future studies. The close interaction between microbial genomics, biosynthetic logic, and natural product metabolomics is critical not only to the clarification of the structures of new chemical entities but also to the final interpretation of genome sequences.

## Conclusion

In conclusion, results of the *M. aurantiaca* sp.01 indicated that this rare actinobacteria species was a potential source for bioactive secondary metabolite production. Genome scanning revealed that at least five antibiotics (similarity more than 15% in the PKS pathway) could be potentially produced from this strain, and kanamycin was confirmed producible in the fermentation experiment by guided MS/MS analysis. The present findings testified that rare actinobacteria from mangroves are important candidates for future pharmaceutical exploration, as they are relatively unexploited, but indeed contain huge biosynthetic potential. Meanwhile, the approach of combining genome mining and guided MS/MS analysis has been shown to be an efficient and effective way to explore the biosynthetic potential of any interested rare actinobacteria under laboratory culture conditions.

## Data Availability Statement

The datasets presented in this study can be found in online repositories. The names of the repository/repositories and accession number(s) can be found in the article/Supplementary Material.

## Author Contributions

DH, CS, KM, and SL conceived the experiments. DH, CS, and SL undertook sampling work. DH, TJ, and GF conducted the experiment. DH, KM, and SL analyzed the results. DH, KM, KL, and SL wrote the manuscript. All authors read and approved the final manuscript.

## Conflict of Interest

The authors declare that the research was conducted in the absence of any commercial or financial relationships that could be construed as a potential conflict of interest.

## Footnotes

1 https://www.ezbiocloud.net/

2 https://antismash.secondarymetabolites.org/#!/about
